# Effect of parenting intervention through “Care for Child Development Guideline” on early child development and behaviors: a randomized controlled trial

**DOI:** 10.1186/s12887-022-03752-x

**Published:** 2022-12-02

**Authors:** Maryam Bemanalizadeh, Negin Badihian, Mehri Khoshhali, Shervin Badihian, Neda Hosseini, Marziye Purpirali, Mansoore Abadian, Omid Yaghini, Seyede Shahrbanoo Daniali, Roya Kelishadi

**Affiliations:** 1 Child Growth and Development Research Center, Research Institute for Primordial Prevention of Non-Communicable Disease, Isfahan University of Medical Sciences, Isfahan, Iran; 2 Department of Child Neurology, Isfahan University of Medical Sciences, Isfahan, Iran; 3 Department of Neurology, Johns Hopkins University School of Medicine, Baltimore, USA; 4 Occupational Therapy Department, School of Rehabilitation Sciences, Isfahan University of Medical Sciences, Isfahan, Iran

**Keywords:** Child development, Child behavior, Parenting education

## Abstract

**Background:**

Several studies showed that parenting intervention programs play a core component in early child development. Considering the limited healthcare resources in developing countries, group-session intervention based on care for child development (CCD) guideline might be cost-effective.

**Methods:**

This randomized controlled trial was conducted at an outpatient public Pediatrics clinic in Isfahan, Iran. We included 210 pregnant women aged 18–45 years in their third trimester and followed their children for 18 months. The intervention group underwent 5 educational group sessions, each lasting for almost 45 minutes. The main outcomes were the children’s development and socio-emotional behavior problems based on Bayley Scales of Infant and Toddler Development-III (BSID-III) at 12 months and the Children Behavior Checklist (CBCL) at 18 months.

**Results:**

Overall, data of 181 children were included in the current study, including 80 in the intervention group and 101 controls. The adjusted median/mean differences between intervention and control groups using median/linear regression were not significant for all BSID-III domains except for median differences for cognitive score based on BSID-III (β (SE): − 4.98(2.31), p:0.032) and mean differences for anxiety/depression score based on CBCL (β (SE): − 2.54(1.27), p:0.046).

**Conclusion:**

In this study, parenting interventions through CCD group sessions were significantly effective on just one subscale of children’s socio-emotional behavior domains based on CBCL and one domain of children’s development based on BSID-III. There might be a ceiling or floor effects for the BSID-III and CBCL assessment, respectively, leaving little room for improvement as almost all children have achieved their full developmental potential in our study.

**Trial registration:**

IRCT20190128042533N2, Date of registration: 16/01/2020, www.irct.ir

**Supplementary Information:**

The online version contains supplementary material available at 10.1186/s12887-022-03752-x.

## Introduction

It is estimated that 250 million children aged under five are at risk of not reaching their full developmental potential all around the world [1–3]. The term “nurturing care” was coined as a core component of what is required to achieve optimal development in the early years of life, when the brain is developing and the child is more responsive to interventions [4]. Parents need to be supported by educational health services to have the necessary knowledge and skills, as they are at the forefront of providing nurturing care for promoting early child development (ECD). WHO/UNICEF provided an intervention package on how to promote ECD entitled “Care for Child Development” (CCD) [5]. It includes the participant manual, counseling cards, facilitator notes, a guide for clinical practice, and a framework for monitoring and evaluation to guide the parents and caregivers on how to engage in play and communication activities that promote motor, cognitive, language, and social-emotional skills and to strengthen responsive caregiving skills by coaching parents and caregivers during play interaction with their child to observe, interpret and appropriately respond to their child’s signals.

Some of the previous studies have suggested that parenting interventions can improve various developmental domains in children, including cognition, language, socio-emotional, and motor abilities [6–10] as well as behavior [11–14]. However, other studies did not support the beneficial effect of such interventions, particularly in those children who are not suffering from any kind of developmental delays [15–17].

However, the majority of studies, specifically on the child’s behavior, were conducted in high-income countries. Evidence showed that parents and children in low-middle income countries or middle-income countries are more susceptible to be exposed to additional risk factors that affect nurturing care and ECD. Low parental education, malnutrition, and fewer early learning opportunities are its commonest determinants. Thus, such support for parenting may be more beneficial in low or middle-resource contexts [3, 18, 19]. Jeong et al. in their systematic review and meta-analysis showed that children in low- or middle-income countries benefit more from parenting programs. They also showed that there are too few studies on social, emotional and behavioral problems. Thus, we decided to assess not only the children’s development but also their socio-emotional behavior problems [19]. Considering the limited healthcare resources in developing countries, we tried to assess the effect of parenting interventions in a cost-effective setting through group sessions CCD intervention on children’s development and socio-emotional behavior problems in Iran as a country located in the Eastern Mediterranean Region.

## Methods

### Study participants

This randomized controlled trial was approved by the ethics committee of Isfahan University of Medical Sciences (ID: IR.MUI.RESEARCH.REC.1397.092), registered in the Iranian Registry of Clinical Trials (IRCT20190128042533N2, Date of registration: 16/01/2020) and has therefore been performed in accordance with the ethical standards laid down in the 1964 Declaration of Helsinki and its later amendments. It was conducted from February 2020 to February 2021 at an outpatient public Pediatrics clinic in Isfahan, Iran. To calculate the sample size, we used the equation for comparing two means with a type I error of 1.96 and a power of 0.90. Assumptions in two groups (including effect size equal to 0.40) were pre-specified based on previously published data [20–22] and the expected effect was hypothesized as a clinically meaningful effect. The sample size was calculated as at least 133 participants in each group. However, due to the COVID-19 pandemic and social restrictions, we stopped the study when 228 participants enrolled. We included Iranian pregnant women aged 18–45 years in their third trimester (days 196 to 280 of pregnancy) who had a healthy fetus based on clinical and ultrasound evaluations, then we followed their children for 18 months. We excluded participants who were absent for more than 2 educational sessions, refused to continue the study, as well as those who had a child with congenital anomaly, birth asphyxia, seizure, or known metabolic or neurological disorders after birth.

### Study design

The study protocol was explained to participants and written informed consent was obtained from participants. Overall, 210 pregnant women were included. Random numbers were generated by random allocation software in binary blocks, and the pediatric neurologist assigned participants into intervention or control groups of equal numbers. The intervention group underwent 5 educational group sessions, each lasting for almost 45 minutes. The sessions were held during the third trimester of pregnancy (the first session), and then 2–6 weeks, 2–6 months, 6–9 months, and 9–12 months after delivery. The educational content of each session was adopted from the Persian version of the Care for Child Development (CCD)-2012 manual (Table 1) [23]. In the intervention group, mothers were trained on how to play and communicate with their children based on the children’s age. We used training materials including educational slides, videos, counseling cards, and toys. Parents engaged in play and communication activities to encourage their children’s learning, according to CCD recommendations. They also learn how to be sensitive to the needs of children and respond appropriately to address their needs through play and communication. The training sessions were held in groups with 20 participants by a pediatric neurologist who was trained by the Iranian Ministry of Health and Medical Education. The control group underwent routine education on child care, suggested by WHO, which is currently implemented in most of the primary health care centers in the country by using pamphlets, as well as general skills training sessions such as sessions on breastfeeding encouragement.

**Table 1:** Topics of parent training sessions based on the Care for Child Development (CCD) manual

Sessions	Elements of care for child development	Session contents
Session 1	**During the third trimester of pregnancy**	Activities to promote playing: • Provide ways for the child to see, hear, move arms and legs freely, and touch the parents. • Describe ways to gently soothe, stroke, and hold the child. • Give the child skin-to-skin contact Activities to promote communicating: • Look into the baby’s eyes and talk to the baby, especially during breastfeeding.
Session 2	**2 week up to 6 weeks**	Activities to promote playing: • Provide ways for the child to see, hear, feel, move freely, and touch the parents. • Provide slowly moving colorful things for the child to see and reach for (sample toys: shaker rattle, big ring on a string). Activities to promote communicating: • Smile and laugh with the child. • Talk to the child. Get a conversation going by copying the child’s sounds or gestures.
Session 3	**2 months up to 6 months**	Activities to promote playing: • Give the child clean, safe household things to handle, bang, and drop (sample toys: containers with lids, metal pot, and spoon). Activities to promote communicating: • Respond to the child’s sounds and interests. • Call the child’s name, and see the child respond
Session 4	**6 months up to 9 months**	Activities to promote playing: • Hide the child’s favorite toy under a cloth or box. See if the child can find it. • Play peek-a-boo. Activities to promote communicating: • Tell the child the names of things and people. • Show the child how to say things with hands, like “bye bye (*sample toy: doll with face).*
Session 5	**9 months up to 1 year**	Activities to promote playing: • Give the child things to stack up, and to put into containers and take out (sample toys: Nesting and stacking objects, containers and clothes clips). Activities to promote communicating: • Ask the child simple questions. • Respond to the child’s attempts to talk. • Show and talk about nature, pictures, and things.

### Outcome measures

The main outcome of the present study was the children’s development at 12 months of age. Moreover, based on our literature review, after the trial registration, we decided to follow up the children’s socio-emotional behavior at 18 months of age. We examined children’s development in 3 key developmental domains of cognition, language, and motor by the Bayley Scales Of Infant and Toddler Development-III (BSID-III) test. It was validated in Persian and the reliability was assessed using Cronbach’s alpha for internal consistency (cognition (*r* = 0.79), language (receptive communication (*r* = 0.76), expressive communication (*r* = 0.81)), motor (fine motor (*r* = 0.80), and gross motor scales (*r* = 0.81))). The standardized Persian version of this questionnaire did not include the other 2 domains of social-emotional and adaptive of the English version of BSID-III [24]. In our study, the test was performed by a certified healthcare provider, who was trained by the Iranian Ministry of Health and Medical Education as one of the staff for performing the BSID-III test in Iran. We also assessed children’s socio-emotional behaviors by CBCL/1½–5, which was developed by Achenbach [25]. It consists of 99 closed items that are rated as not true (score 0), somewhat/sometimes true (score 1), or very true/often true (score 2) and one open-ended item. Mothers completed the Persian version of CBCL [26, 27], normalized and translated by Mohammad Esmaeel [28]. In addition to a total problem score, CBCL contains 2 broadband scales (externalizing problems and internalizing problems) and 8 narrowband syndrome scales including emotionally reactive, anxiety/depression, somatic complaints, withdrawal, sleep problems, attention problems, aggressive behavior, and other problems. The latter involves a range of heterogeneous problems including jealousy, disobedience, food refusal, overeating, being overweight, nail-biting, nightmares, etc. DSM-V scales include affective problems, anxiety problems, pervasive developmental problems, attention-deficit/ hyperactivity problems, and oppositional defiant problems.

Good test-retest reliability was found with correlation coefficients ranging from 0.37 to 0.91 for each item. The average test-retest reliability coefficients for the total competence score and the total problems score were 0.80 and 0.79, respectively [29]. In the test re-test analyses conducted by Achenbach et al. [29] with a two-week interval, a test-retest coefficient of 0.87 was reported for behavioral problems.

Sociodemographic data were collected from the mothers in both groups at the time of enrollment. The number of children, educational level of parents, parental ages, their income, history of paternal smoking, infant’s gender, Apgar scores, gestational age, and delivery type were collected.

### Statistical analysis

Continuous variables were expressed as mean ± standard deviation (SD), and categorical data as numbers (percentages). We assessed the normality of data by statistical (by the Kolmogorov–Smirnov test) and graphical tests (Box- plots). Comparisons between means of continuous variables in the intervention and control groups were performed using an independent Student t-test. We used the Chi-square test to assess the difference in the distribution of the categorical variables between intervention and control groups. We calculated the adjusted median and the mean differences between the intervention and control groups by median regression and linear regression models, respectively. The regression models were performed for each domain of BSID-III assessment and CBCL, separately. The models were adjusted for maternal and paternal age and educational levels, paternal cigarette smoking, preterm labor, type of delivery, and child’s gender. We selected the covariates based on a review literature. All analyses followed per-protocol principles. Data analyses were performed using the statistical software STATA 12.0 (STATA Corp, College Station, Texas, USA). *P*-values of less than 0.050 were considered statistically significant**.**

## Results

**Fig. 1:**
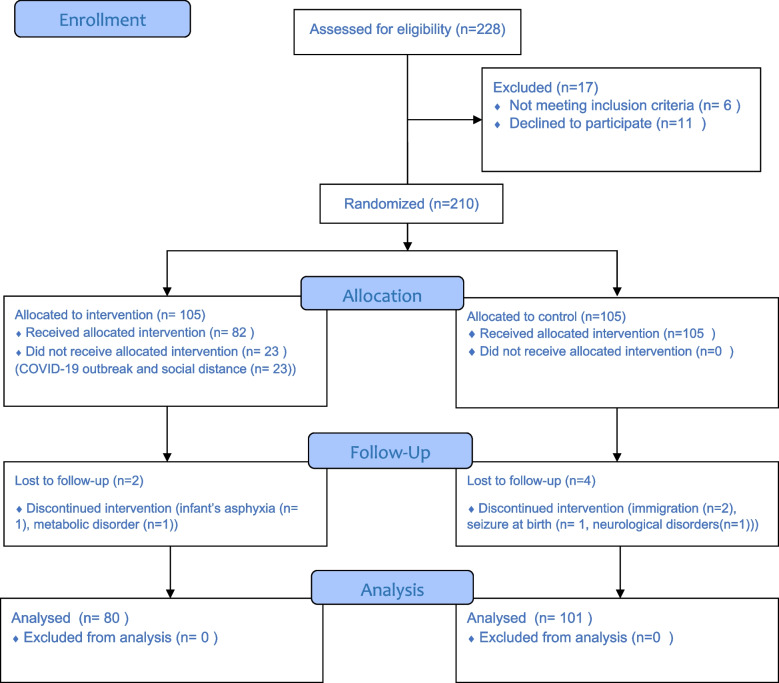
Flow diagram of the study

Among 210 participants, data of 181 children were complete and used in the present study (80 children in the intervention group and 101 children in the control group). We lost 23 participants in the intervention group because of the COVID-19 outbreak and social distance. Moreover, we lost to follow-up 2 and 4 children in the intervention and control groups, respectively (Fig. 1).

Table 2 shows the maternal, parental, and child characteristics by the groups. Maternal and paternal age in the control group was significantly lower than intervention group (*p* < 0.05), while there were no significant differences across maternal and paternal education, income, paternal smoking, gender, gestational age and delivery type between groups.

**Table 2:** Parents and children’s characteristics

Parents and children's charecteristics		Intervention	Control	*p* ^a^
Parents				
Maternal age, mean ± SD		31.99 ± 4.72	29.48 ± 5.17	0.001
Paternal age, mean ± SD		37.23 ± 8.49	34.07 ± 5.09	0.002
Maternal education, n(%)				
	Secondary school and less	8 (10.1%)	13(12.9%)	0.847
	High school or diploma	35(44.3%)	44(43.6%)	
	University education	36(45.6%)	44(43,.6%)	
Paternal education, n(%)				
	Secondary school and less	25 (31.6%)	33(33.7%)	0.740
	High school or diploma	34(43%)	45(45.9%)	
	University education	20 (25.3%)	20(20.4%)	
Income, n(%)				
	Low	14 (17.5%)	28 (27.7%)	0.090
	Moderate	54 (67.5%)	66 (65.3%)	
	High	12 (15%)	7 (6.9%)	
Paternal smoking				
	No	68 (85%)	85(84.2%)	0.876
	Yes	12 (15%)	16 (15.8%)	
Infants				
Gender, n(%)	Girl	41 (51.3%)	53 (52.5%)	0.870
Gestational age, n(%)	< 37 week	1 (1.3%)	5(5%)	0.167
Delivery Type, n(%)	Cesarean	41(51.3%)	56(55.4%)	0.268

*Abbreviations*: *SD* Standard deviation. ^a^ Results are shown based on the student t-test or chi-square analysis

### Neurodevelopment outcomes

The Kolmogorov–Smirnov test for the cognitive score, language score, and motor score of BSID III, and all CBCL scales were significant (*p* < 0.05), thus the distribution of these variables was not normal.

### BSID-III assessment

The means/medians for all scores were more than 100 in both intervention and control groups. The adjusted median differences between intervention and control groups using median regression were not significant for all BSID-III domains except for cognitive score (β (SE): − 4.98(2.31), p:0.032). We also provided the results of the linear regression and we found no significant differences in cognitive, language and motor scores of BSID-III between the two groups (Table 3).

**Table 3:** Results of BSID-III in the intervention and control groups at 12 months of age

BSID-III assessment	Intervention (*n* = 80)	Control (*n* = 101)	SMD	Unadjusted median/mean differences		Adjusted median/mean differences ^e^	
				β (SE)	***p***	β (SE)	***p***
Cognitive score	105 (100–115) ^a^	110 (100–120)	–	-5(1.69) ^c^	0.004	−4.98(2.31)	0.032
	108.06 ± 11.12 ^b^	110.56 ± 12.95	−0.205	−2.49(1.83) ^d^	0.175	−2.79(1.93)	0.150
Language score	112(103–118)	112(103–118)	–	0(1.96)	1	− 0.39(2.35)	0.870
	110.81 ± 11.02	111.15 ± 10.63	−0.031	−0.34(1.62)	0.835	−0.63 (1.74)	0.717
Motor score	103(97–110)	107(91–112)	–	−4(2.55)	0.118	−0.32(2.56)	0.899
	102.48 ± 11.64	103.26 ± 13.65	−0.061	−0.78(1.93)	0.686	−0.46 (2.12)	0.828

*Abbreviations*: *BSID-III* Bayley Scales of Infant and Toddler Development-III, *SMD* Standardized mean difference, *SE* Standard error, *IQR* Interquartile range, *SD* Standard deviation. ^a^ Results are shown as median (IQR). ^b^ Results are shown as mean ± SD. ^c^ Results are shown based on median regression. ^d^ Results are shown based on linear regression. ^e^ Adjusted by maternal and paternal age and educational levels, gestational age, types of delivery, paternal smoking (cigarette), and child’s gender

### CBCL assessment

The adjusted median differences between intervention and control groups using median regression were not significant for any CBCL syndrome scales. The adjusted mean differences between intervention and control groups using linear regression were not significant for all CBCL syndrome scales except for anxiety/depression score(β (SE): − 2.54(1.27), p:0.046) (Table 4).

**Table 4:** Results of CBCL in the intervention and control groups at 18 months of age

CBCL syndrome scales	Intervention (*n* = 80)	Control (*n* = 101)	SMD	Unadjusted median/mean differences		Adjusted median/mean differences ^e^	
				β (SE)	***p***	β (SE)	***p***
Emotionally reactive	42(35–46) ^a^	42(35–46)	–	0(0.67) ^c^	1	0(1.22)	1
	40.98 ± 6.41^b^	42.61 ± 6.90	−0.245	− 1.64(1) ^d^	0.103	− 0.88(1.06)	0.410
Anxiety/depression	42(38–53)	46(42–53)	–	−4(2.11)	0.059	−2.51(2.04)	0.221
	44.15 ± 8.28	46.82 ± 7.70	−0.336	−2.67(1.19)	0.026	−2.54(1.27)	0.046
Somatic complaints	35(35–44)	40(35–44)	–	−5(1.58)	0.002	−1.04(1.64)	0.528
	39.49 ± 5.75	40.87(6.64)	−0.221	−1.38(0.94)	0.142	−1.22 (0.99)	0.220
Withdrawn	40(40–40	40(40–40)	–	–	–	–	–
	40.65 ± 2.56	41.06 ± 2.94	−0.147	−0.41(0.42)	0.326	−0.17(0.44)	0.703
Sleep problems	48(48–52	52(44–57)	–	−4(0.87)	< 0.001	−1.55(0.92)	0.094
	49.34 ± 5.75	51.45 ± 9.85	−0.254	−2.11(1.24)	0.091	−1.78 (1.30)	0.173
Attention problems	35(35–41)	41(35–46)	–	−6(1.91)	0.002	−1.51(1.47)	0.305
	39.48 ± 7.47	41.40 ± 7.67	−0.253	−1.92(1.14)	0.92	−1.24(1.21)	0.308
Aggressive behavior	40(37–43)	40(37–46)	–	0(0.99)	1	−0.32(1.29)	0.805
	40.09 ± 6.04	40.86 ± 8.30	−0.105	−0.77(1.11)	0.485	−0.98 (1.21)	0.416
Other problems	42(40–48)	44(41–51)	–	−2(1.21)	0.1	−1.32(1.47)	0.372
	44.34 ± 7.64	45.84 ± 7.25	−0.203	−1.50(1.11)	0.177	−0.92 (1.18)	0.438
Internalizing behaviors	38(36–41)	40(37–43)	–	−2(0.87)	0.023	−0.81(0.88)	0.362
	39.30 ± 4.37	40.97 ± 4.74	−0.364	−1.67(0.69)	0.016	−1.24 (0.72)	0.089
Externalizing behaviors	38(35–43)	39(37–48)	–	−1(1.14)	0.381	−0.86(1.35)	0.524
	40.69 ± 7.01	42.52 ± 7.53	−0.252	−1.84(1.09)	0.094	−1.28 (1.17)	0.176
Total score	43(39–51)	47(41–56)	–	−4(1.67)	0.018	−2.75(1.96)	0.162
	45.81 ± 9.79	49.16 ± 10.01	−0.338	−3.35(1.84)	0.025	−2.42 (1.57)	0.125

*Abbreviations*: *CBCL* Child Behavior Checklist, *SMD* Standardized mean difference, *SE* Standard error, *IQR* Interquartile range, *SD* Standard deviation. ^a^ Results are shown as median (IQR). ^b^ Results are shown as mean ± SD. ^c^ Results are shown based on median regression. ^d^ Results are shown based on linear regression. ^e^ Adjusted by maternal and paternal age and educational levels, income, gestational age, types of delivery, paternal smoking (cigarette), and child’s gender

## Discussion

In the present study, in general, educating mothers about the CCD recommendations in a group setting did not lead to a better developmental and behavioral status of children in the intervention group compared to the control group. There were slight significant differences between groups in the children’s behavior subscales, including anxiety/depression in the syndrome-oriented scale and cognitive score based on BSID-III assessment.

To the best of our knowledge, this is the first study in a developing country in a group setting, focusing on the socio-emotional behaviors as well as some other developmental domains in children in a parenting intervention program. Although parenting programs are well evaluated in developing countries, focusing on the socio-emotional behavior problems as well as some other developmental domains were less considered, particularly in a group setting, which could be more feasible and accessible in health care system. Most studies in low or middle-income countries have assessed some other developmental outcomes, focusing less on the children’s socio-emotional behavior problems [8, 30–34]. As noted above, we conducted CCD sessions for parents in a group setting. Although our study found no effects on the Bayley measures of development except for cognitive score, other studies using group delivery did find significant effects [16, 32, 35, 36]. Possibly our lack of effects is due to the relatively high Bayley scores of our children at baseline, leaving little room for improvement, and the fewer sessions we provided compared to other studies. Since cost analysis revealed that the group-based model required a quarter of the costs compared to home visiting [19, 37], further studies are needed to test the effectiveness and evaluate the implementation processes of the CCD parenting program using a group delivery format. Our finding that children’s socio-emotional problem behaviors were lower in the intervention group in one subscale was consistent with the pooled results of a meta-analysis that showed parenting interventions reduce behavioral problems (SMD = − 0.13, 95% CI: − 0.18, − 0.08, *p* < 0.001, *p* < 0.001) [19]. We found a significant effect of parenting intervention on cognitive development assessed by BSID-III, similar to Jeong et al.’s meta-analysis on children’s cognitive development (SMD = 0.41, 95% CI: 0.29 to 0.53, *p* < 0.001), however our results were not consistent with the results of Jeong et al.’s meta-analysis for language development (SMD = 0.35, 95% CI: 0.21 to 0.48, *p* = 0.02), and motor development (SMD = 0.26, 95% CI: 0.16 to 0.36, *p* = 0.03) [19]. The inconsistent results might arise from few training sessions, the high mental development scores of included children as well as the small sample size in our study.

Identifying primary preventative interventions that would be effective and feasible-delivered is helpful to decision-makers. It can shed lights in reducing the burden of neurodevelopmental disorders and behavioral problems in low- and middle-income countries as it highlights where resources should be allocated [15].

### Limitations and strengths

There are some limitations to the present study that may have affected our findings. First and foremost, the main limitation is that children’s developmental scores based on BSID-III were probably at the ceiling, leaving little room for improvement. BSID scores are from a norm-referenced assessment of early childhood development. Although the selection of neurotypical children might cause an increase in the mean scores, the differences in cultural, environmental, and genetic factors might also influence development. Our sample may not be representative of Iran and may be dissimilar to other parenting evaluations; for example, almost half of our mothers were university-educated. Second, too little time was spent in sessions due to the restrictions we had during the COVID-19 crisis. Third, although we approached to follow up all participants in, we lost a considerable number due to the COVID-19 outbreak. Many mothers in the intervention group were not willing to participate in the sessions anymore because of social distancing rules. It is well documented that parents who accept to participate in parenting programs are different than the ones who do not participate [38]. This issue affects the representability of the study sample, so the result should be interpreted with caution. Fourth, we were not able to assess parents’ knowledge before and after the intervention, and their adherence to CCD instructions due to the limited resources. Measuring parenting practices assessing by some standard tools such as the HOME [39] would also benefit our findings. Fifth, in this study, we just involved mothers in group sessions. Despite the observational evidence that fathers are critically important for ECD [40], we could not involve fathers in our parenting program due to their work time as well as low compliance for the study. The last limitation was that caregivers in the intervention group could have been biased in reporting on their child’s behavior in the CBCL assessment. Therefore, this should be considered when interpreting the findings of the present study. Despite these limitations, this study has focused on children’s behavior during infancy and early childhood. Most parenting programs for behavioral development to date have been commonly delivered to preschool-aged children rather than young children during the earliest years of life and our information on early childhood was scarce [18, 41–43]. Moreover, we represented the child’s socio-emotional behavior through a valid and reliable tool, while several previous studies reported no details regarding the reliability or validity of the measurement tool for behavioral problems [19]. Another strength of the current study is its novelty in developing countries and the first study of its kind in the region.

## Conclusion

In the present trial, delivering parenting interventions through CCD group sessions had significant effects on just one subscale of children’s behavior domains based on CBCL assessment, and one domain of children’s development based on BSID III. There might be ceiling or flooring effects for the BSID-III and CBCL assessment, leaving little room for improvement as almost all children achieved their full developmental potential in our study. There is little evidence to support that CCD is critical for improving children’s development and socio-emotional behaviors. Moreover, the feasible method of its delivery to a large population, especially in developing countries, remains to be determined. Longitudinal studies on a large sample of children are necessary in this regard.

## Supplementary Information

**Additional file 1.** 

## Data Availability

The datasets used and/or analyzed during the current study are available from the corresponding author on reasonable request.

## Abbreviations

BSID-III: Bayley scales of infant and toddler development-III
CBCL: Children behavior checklist
CCD: Care for child development
ECD: Early child development
SD: Standard deviation
SE: Standard error
